# Biologically Effective Dose and Dose Rate in Gamma Knife Radiosurgery for Trigeminal Neuralgia: A Systematic Review and Meta-Analysis

**DOI:** 10.1016/j.adro.2025.101932

**Published:** 2025-10-23

**Authors:** Jane Jomy, Ke Xin Lin, Radha Sharma, Rachel Lu, Sanchit Kaushal, Anna T. Santiago, Dana Keilty, David Shultz, Catherine Coolens, Michael D. Cusimano, Gelareh Zadeh, Mojgan Hodaie, Suneil K. Kalia, Farshad Nassiri, Ying Meng, Derek S. Tsang, Michael Yan

**Affiliations:** a MD Program, Temerty Faculty of Medicine, University of Toronto, Toronto, Ontario, Canada; b Department of Biostatistics, University Health Network, Toronto, Ontario, Canada; c Radiation Medicine Program, University Health Network, Toronto, Ontario, Canada; d Division of Neurosurgery, Unity Health Toronto, Toronto, Ontario, Canada; e Neurosurgery, Mayo Clinic, Phoenix, Arizona; f Division of Neurosurgery, University Health Network, Toronto, Ontario, Canada; g Division of Neurosurgery, Sunnybrook Health Sciences Centre, Toronto, Ontario, Canada

## Abstract

**Purpose:**

Gamma Knife radiosurgery (GKRS) is used in the treatment of trigeminal neuralgia (TN) to deliver precise, focused ionizing radiation to the trigeminal nerve, thereby reducing its ability to transmit pain signals. Understanding the impact of unique radiobiological parameters, such as the biologically effective dose (BED) and dose rate, in GKRS is essential to optimize treatment protocols and ensure predictable therapeutic outcomes. We conducted a systematic review and meta-analysis to evaluate how BED and dose rate impact GKRS effectiveness and toxicity.

**Methods and Materials:**

We searched medical and health care databases from inception to May 19, 2024, for publications reporting on the impact of GKRS BED and/or dose rate on TN outcomes. Following 2 rounds of screening conducted in duplicate, we used random-effects meta-analysis and meta-regression to examine the association between dose rate and pain relief.

**Results:**

Of 6950 citations identified, 8 publications reported data on BED and dose rate association with GKRS patient outcomes in TN. Eight cohorts reported on 2596 patients in 6 countries. The “beam-on” time ranged from 27 to 171 minutes, and the prescription dose ranged from 62.5 to 95 Gy. The median BED_2.47_ was 2105 Gy (range, 1968-2675), the median dose rate was 2.2 Gy/min (range, 2.06-2.81), and the median maximum brainstem dose was 20.7 Gy (range, 14.8-34.7). The meta-analysis suggested that higher dose rates may be associated with higher rates of pain relief (relative risk, 1.36 [95% CI, 1.10-1.67]; *P* = .005). Meta-regression demonstrated a nonsignificant relationship between dose rate and pain relief, with an estimated 26% increase in the likelihood of pain control for each 1 Gy/min increase in the median dose rate (β, 0.26 [95% CI, −1.09, 1.60]; *P* = .71).

**Conclusions:**

Higher dose rates in GKRS may be associated with better pain relief in TN. Dose rate should be considered in the treatment of TN when using GKRS.

## Introduction

Trigeminal neuralgia (TN) is characterized by facial pain caused by irritation or compression of the trigeminal nerve, with type 1 (classical) pain being sharp, intermittent, and triggered by stimuli, and type 2 (atypical) being constant and dull.^1^ Medications, such as anticonvulsants, are commonly used to reduce and prevent TN symptoms.^2^ However, in some patient populations, medications may not be effective, with common explanations including an etiology related to multiple sclerosis or tumor mass effect.^3^ Treatment options for such refractory TN include microvascular decompression, rhizotomy, and stereotactic radiosurgery.^4^ Gamma Knife radiosurgery (GKRS), a specific type of stereotactic radiosurgery, focuses ionizing radiation emitted from 192 Cobalt-60 (Co-60) sources on the afflicted trigeminal nerve root, causing nerve lesioning that reduces its ability to transmit pain signals.^5^

It is well established in the literature that dose prescription is only one component of many radiobiological factors that contribute to treatment effectiveness and toxicity.^6^ The term “biologically effective dose” (BED) was proposed in 1989, based on linear-quadratic cell survival in radiobiology, to quantify the biological effect of a radiation therapy treatment.^7^ The BED formula incorporates changes in dose rate, dose prescription, and overall treatment time.^6^^,^^8^ Dose rate represents the amount of dose delivered over time, expressed in units of Gy/min. As the dose rate is increased, the fraction of cells killed by a given dose increases. Dose rate is influenced by the size of the collimator, the size of the patient’s head, and blocking.^9^ A new Co-60 source has a higher dose rate and, in turn, an increased estimated BED for GKRS treatments for the same physical dose prescription.^9^ Currently, most GKRS installations replace Co-60 sources every 5 to 7 years, leading to a range of dose rates from 3.7 Gy/min for new sources to 0.9 Gy/min for sources after 2 half-lives.^10^ This variability raises significant concerns regarding the radiobiological consistency and efficacy of GKRS over a source’s lifetime.

In comparison with dose prescription, the impact of both BED and dose rate on treatment effectiveness and toxicity is not well-characterized. Understanding the impact of radiobiological factors is essential for optimizing treatment protocols and ensuring predictable therapeutic outcomes while minimizing radiation-related risks. We conducted a systematic review and meta-analysis to evaluate how BED and dose rate impact GKRS treatment effectiveness and toxicity.

## Methods and Materials

This systematic review was conducted in adherence to the 2020 Preferred Reporting Items for Systematic Reviews and Meta-Analysis guidelines.^11^ We registered our protocol with PROSPERO (the International Prospective Register of Systematic Reviews): CRD42024549214. This study was exempt from research ethics review.

### Eligibility criteria

We included any primary studies on the impact of GKRS BED and/or dose rate on treatment effectiveness and/or toxicity in TN. BED refers to the dose of radiation that accounts for the biological effect of the radiation on tissues, considering both the total dose and the fractionation scheme.^6^ It is used to estimate tumor control and normal tissue toxicity based on the delivered radiation dose, considering the different response rates of various tissues to radiation. The formula used to calculate BED may vary across the included studies because of new radiobiological models that have emerged in the field of radiation oncology. Nilsson and Thames^12^ and Thames^13^ developed a biological model to calculate BED, $BED_{\alpha /\beta }=d\left(1+\frac{gd}{\alpha /\beta }\right)$, where α/β is a tissue-specific constant, *d* is the total dose, *g* is the continuous repair factor and is equal to $2[\mu t-\;1+e\left(-\mu t\right)/{\left(\mu t\right)}^{2}$, and μ is the recovery constant and is equal to $\ln2/T_{1/2}$. Subsequently, Hopewell et al^8^ developed the equation: $BED_{\alpha /\beta }=D_{T}+\frac{1}{\alpha /\beta }\left[\frac{\varphi \left(\xi ,\mu_{1}\right)+\varphi \left(\xi ,\mu_{2}\right)}{1+c}\right]{D^{2}}_{T}$, where α/β is a tissue-specific constant, *D_T_* is the total dose, φ(ξμ)is a complex function of the protocol and repair rates, and *c* is a partition coefficient. Preclinical studies and non-English articles were excluded. We did not exclude abstracts.

### Information sources and searches

Systematic, structured literature searching was conducted by a trained clinical epidemiologist (J.J.). Several health care and medical databases, including MEDLINE, Embase, the American Psychological Association PsycInfo, and Web of Science, were systematically searched from inception to May 19, 2024, using the search strategy in Appendix E1. To identify any potentially missing studies, the searches were supplemented by a manual search of the reference lists of retrieved papers and review articles.

### Study selection

Five independent reviewers (J.J., R.L., K.X.L., R.S., and S.K.) performed 2 levels of screening for eligible studies using Covidence (Veritas Health Innovation, www.covidence.org). All disagreements between reviewers were identified in Covidence and resolved through joint discussion until mutual agreement. A third reviewer could be involved in adjudication. All studies removed at either screening level had a documented reason for exclusion, in accordance with the Preferred Reporting Items for Systematic Reviews and Meta-Analysis guidelines.

### Data extraction

A standardized data extraction form was developed jointly by 2 investigators (J.J. and M.Y.), and consensus exercises were conducted prior to data extraction. Data extraction was conducted by 2 investigators (J.J. and K.X.L.), and any disagreements were resolved through discussion until consensus was reached. The following data were extracted from the included literature: study characteristics (country, study design, number of recruitment sites, number of patients, number of female patients, median age, and follow-up duration), clinical characteristics (type of TN, previous interventions, medications, duration of symptoms, time since diagnosis, pain scores, pain distribution, and right-sided pain), and radiosurgery parameters (number of isocenters, median beam-on time, prescription dose, BED, dose rate, and maximum brainstem dose).

### Risk of bias assessment

Using criteria proposed by the Clinical Advances through Research and Information Translation group at McMaster University,^14^ 2 investigators (J.J. and K.X.L.) independently and in duplicate assessed the risk of bias of all included studies. The criteria included selection bias, control for confounding variables, validity of outcome assessment(s), and infrequent missing data (<20%). We categorized a study as high risk of bias if any 1 criterion met the threshold for high risk of bias. Risk of bias assessments are presented using the *robvis* software (https://mcguinlu.shinyapps.io/robvis/) in Fig. E1.^15^ This criterion was applied only to full-text articles; any abstracts received a “not applicable” score across all domains for completeness.

### Data analysis

We summarized the study population descriptively. The risk ratio (RR) was estimated as a measure of the effect of high- versus low-dose-rate (Gy/min) on patient outcomes. RR was estimated using available frequency and/or proportion data. A random-effects meta-analysis was performed using the inverse-variance method to weight each study, and statistical significance was reported for the overall effect estimate.^16^ Heterogeneity was measured using the Tau^2^, χ2 (Q-statistic), and $I^{2}$ values.^17^ Publication bias was evaluated using the trim-and-fill method and a funnel plot to obtain an adjusted estimate of the overall effect. Meta-regression was performed to explore whether the median dose rate (Gy/min) contributed to between-study heterogeneity. Statistical analysis was performed using the *meta* library in R version 4.4.1 (R Foundation for Statistical Computing). A 2-sided *P* value < .05 was considered statistically significant.

### Certainty of evidence

We used the Grading of Recommendations Assessment, Development and Evaluation (GRADE) approach to appraise the certainty of evidence.^18^ With this approach, evidence begins as high certainty but can be downgraded to moderate, low, and very low because of risk of bias, indirectness, imprecision, inconsistency, or publication bias. High certainty indicates high confidence that the true effect lies close to the estimate of the effect; moderate certainty indicates moderate confidence that the true effect is likely to be close to the estimate, though there is a possibility that it is substantially different; low certainty indicates that the true effect may be substantially different from the estimate; and very low certainty indicates that the true effect is likely to be substantially different from the estimate of the effect. Per GRADE, the overall certainty of evidence per meta-analysis is presented in tabular format in Table E1.^18^, ^19^, ^20^, ^21^, ^22^, ^23^, ^24^, ^25^, ^26^

## Results

**Figure 1:**
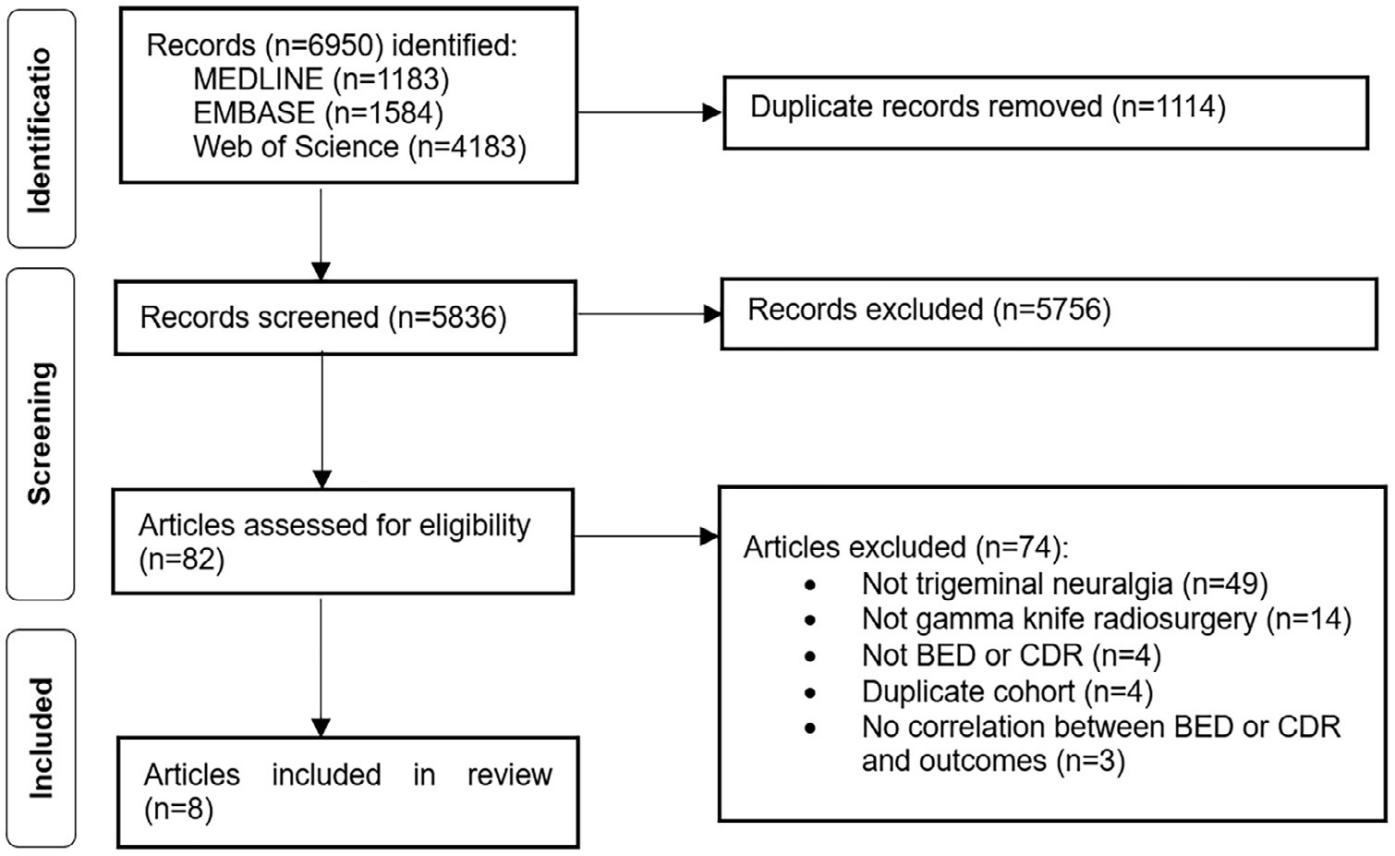
Preferred Reporting Items for Systematic Reviews and Meta-Analysis flow diagram of the study selection. *Abbreviations:* BED = biologically effective dose; CDR = cumulative dose rate.

Our search identified 6950 citations, of which we reviewed 80 full-text articles. Of these, 8 publications were included (Fig. 1).

### Study characteristics

The baseline characteristics of the included studies are described in Table 1. The 8 publications reported on 2596 patients in 6 countries.^19^, ^20^, ^21^, ^22^, ^23^, ^24^, ^25^, ^26^ Overall, the studies included a median of 216 patients (range, 61-871) per study, with a median age of 67 years (range, 59-70). Half of the studies recruited only patients with type 1 TN,^19^^,^^24^, ^25^, ^26^ while 2 studies included those with type 1, type 2, and TN secondary to multiple sclerosis.^20^^,^^23^ Some studies included patients who had undergone previous surgical intervention (ie, microvascular decompression),^19^^,^^23^ while others excluded them from participation.^24^, ^25^, ^26^

**Table 1:** Baseline characteristics of the included studies

Author year	Country	Study design	Recruitment sites	Recruitment range	Total patients, N	Female, (%)	Age (y) at time of GKRS, median (range)	Follow-up duration (mo), median (range)	Types of TN	Previous surgery, (%)	Right-sided pain, (%)
Arai 2010^19^	USA	Retrospective	1	1994-2005	165	62	70 (36-89)	26 (maximum, 139)	Type 1	28	54
Balamucki 2006^20^	USA	Prospective	1	1999-2004	239	63	65 (22-90)	17 (0.7-59)	Type 1 or 2, TN-MS	-	-
Barzaghi 2021^21^	Italy	Retrospective	1	2009-2018	112	66	66 (28-92)	58 (12-126)	-	-	47
Patel 2005^22^	USA	Retrospective	1	1998-2000	61		-	30 (15-49)	-	-	-
Yang 2022^23^	USA	Retrospective	1	2006-2018	192	60	68* (67-69)	-	Type 1 or 2, TN-MS	13	59
Warnick 2024^24^	USA, Canada, Dominican Republic, India, Turkey, and the Czech Republic	Retrospective	13	-	871	60	68 (23-91)	21 (6-156)	Type 1	0	60
Tuleasca 2020^25^	France	Retrospective	1	1997-2010	408	-	-	Minimum: 12	Type 1	0	-
Tang 2025^26^	China	Retrospective	3	2012-2020	548	51	59 (55-62)	Minimum: 36	Type 1	0	52

*Abbreviations:* GKRS = Gamma Knife radiosurgery; TN-MS = trigeminal neuralgia in multiple sclerosis.

⁎ Mean age at time of GKRS.

All but 1 of the studies were retrospective cohort studies.^19^^,^^21^, ^22^, ^23^, ^24^, ^25^, ^26^ Four studies reported follow-up duration, ranging from less than 1 month to 13 years.^20^, ^21^^,^^24^ Nearly all studies recruited patients from a single country (7/8; 88%), while the remaining study had 13 recruitment sites across the United States, Canada, the Dominican Republic, India, Turkey, and the Czech Republic.^24^ Most studies recruited patients from the United States (5/8; 63%).^19^^,^^20^^,^^22^, ^23^, ^24^ Other studies included patients from Italy,^21^ France,^25^ and China.^24^

Radiosurgical parameters are summarized in Table 2.^8^^,^^12^^,^^13^^,^^19^, ^20^, ^21^, ^22^, ^23^, ^24^, ^25^, ^26^ Nearly all studies used a single isocenter, while 2 studies included patients who underwent GKRS with 1 or 2 isocenters.^20^^,^^24^ The size of the shots was mostly 4 mm,^19^, ^20^, ^21^^,^^23^, ^24^, ^25^, ^26^ with 1 study also including 8 mm shots.^20^ The “beam-on” time (treatment time) ranged from 27 to 171 minutes across the 4 studies that reported this parameter,^19^^,^^23^, ^24^, ^25^, ^26^ with a prescription dose of 62.5 to 95 Gy.^19^, ^20^, ^21^, ^22^, ^23^, ^24^, ^25^, ^26^ The median BED_2.47_ was 2105 Gy (range, 1968-2675),^20^^,^^23^, ^24^, ^25^, ^26^ and the median dose rate was 2.2 Gy/min (range, 2.06-2.81).^19^, ^20^, ^21^, ^22^, ^23^, ^24^ The median maximum brainstem dose was 20.7 Gy (range, 14.8-34.7).^21^^,^^23^^,^^24^^,^^26^

**Table 2:** Radiosurgical parameters of the included studies

Author year	Isocenters	Size of isocenter (mm)	Beam-on time (min), median (IQR) or range	Prescription dose (Gy) or range	BED calculation	BED (Gy), median (IQR) or range	Dose rate (Gy/min), median (IQR) or range	Maximum brainstem dose (Gy), median (IQR)
Arai 2010^19^	1	4	26.73-95.11	Maximum: 80	-	-	2.06 (1.21-3.74)	-
Balamucki 2006^20^	1 or 2	4, 8	-	80-90	Nilsson and Thames^12^ and Thames^13^	1782-3479	2.001-3.627	-
Barzaghi 2021^21^	1	4	-	Maximum: 80 (70-90)	-	-	2.2 (1.4-3.3)	10.6 (10.3-10.9)
Patel 2005^22^	1	-	-	75 (70-80)	-	-	1.62 (1.51-1.79) 3.43 (3.21-3.66)	-
Yang 2022^23^	1	4	40 (28-69)	80	Nilsson and Thames^12^ and Thames^13^	1962 (1703-2104)	2.13 (1.19-2.86)	18.9
Warnick 2024^24^	1	4	43.2 (25-170.5)	80 (62.5-95)	Hopewell et al^8^	1973.9 (1253.1-2530.2)	-	22.4 (1.7-75.8)
Tuleasca 2020^25^	1	4	25-135	75-97.9	Hopewell et al^8^	1535-2675	-	-
Tang 2025^26^	1 or 2	4	Minimum: 32.09 (26.81-39.64)	Maximum: 83-90	Hopewell et al^8^	2719.81 (2665.36-2752.84)	2.68 (2.22-3.17)	47 (34-58)

*Abbreviations:* BED = biologically effective dose.

### Dose rate

GKRS dose rate in TN was reported for 882 patients across 5 studies.^19^, ^20^, ^21^, ^22^, ^23^ The median dose rate was 2.2 Gy/min (range, 1.84-2.47).

#### Pain relief

**Figure 2:**
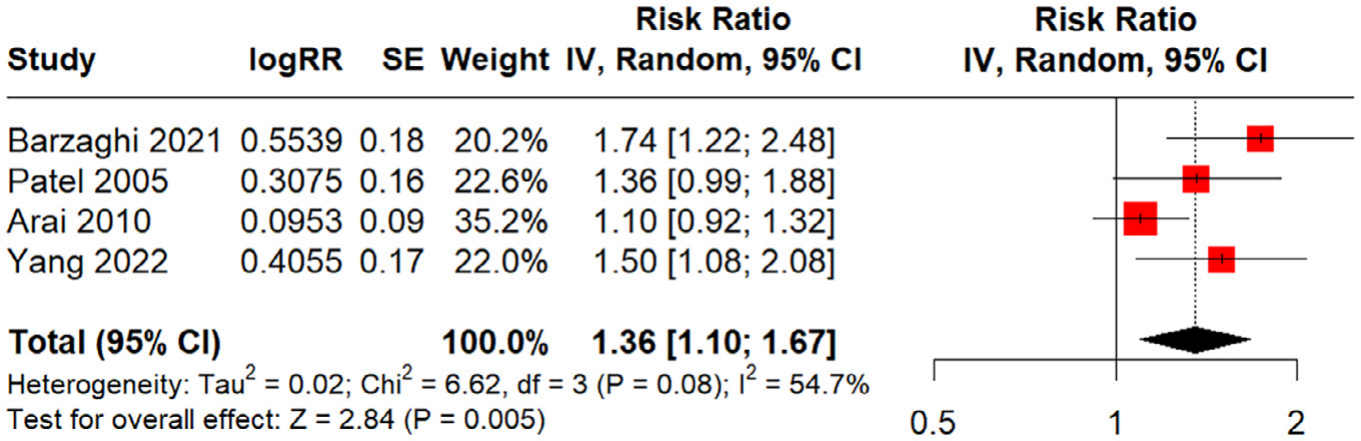
Meta-analysis forest plot for pain relief among trigeminal neuralgia patients; risk ratio (RR) for pain relief in relation to higher dose rates. *Abbreviations:* IV = inverse variance.

Four studies assessed pain relief in relation to low- versus high-dose-rates.^19^^,^^21^, ^22^, ^23^ Three of the 4 studies set a threshold to dichotomize low- and high-dose-rates. Low-dose-rate was defined as <2.5 Gy/min^21^^,^^23^ or <2.05 Gy/min.^19^ One study reported a median of 1.62 Gy/min for the low-dose-rate group and 3.43 Gy/min for the high-dose-rate group.^22^ The estimated overall effect (RR) of dose rate on pain relief was 1.36 (95% CI, 1.10-1.67), *P* = .005 (Fig. 2), indicating greater pain relief with higher dose rates.

We have very low certainty that this estimated effect is close to the true effect based on the GRADE assessment (Table E1). Heterogeneity measures (Tau^2^ = 0.02, I^2^ = 54.7%) and the test for heterogeneity (χ2 [Q] = 6.62, *P* = .08) indicated that moderate heterogeneity may exist among the 4 studies. The adjusted estimate of the overall effect (RR) using the trim-and-fill method to account for publication bias was 1.18 (95% CI, 0.93-1.49) (*P* = .18), with heterogeneity values of Tau^2^ = 0.06, I^2^ = 69.2%, and χ2 (Q) = 16.22 (*P* = .006), indicating moderate heterogeneity (funnel plot available in Fig. E2). In addition to the 4 studies included in the quantitative synthesis, which could not be pooled, Balamucki et al^20^ also investigated the impact of dose rate on the rate of pain control and the degree of pain relief, demonstrating a nonsignificant relationship (*P* > .05).

**Figure 3:**
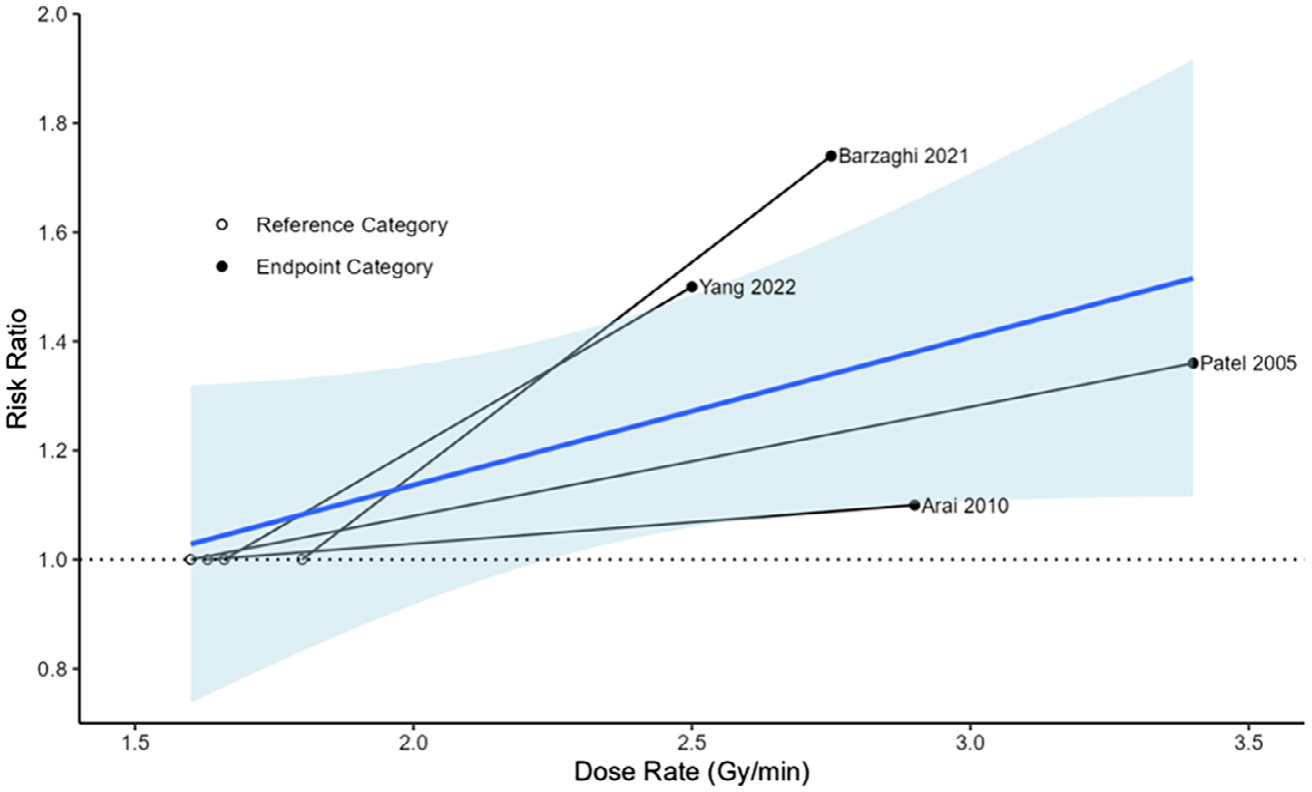
Relationship of pain relief and dose rate among patients in the 4 trigeminal neuralgia studies. Data points represent the median dose rate values assigned to the endpoint category (high-dose-rate) and the reference category (low-dose-rate), along with the corresponding risk ratio relative to the reference category. A line of best fit describes the average relationship.

We conducted a meta-regression to investigate the relationship between RR and median dose rate for the high- (endpoint) and low-dose-rate (reference) categories. This relationship was not significant (Fig. 3), with an estimated 26% increase in the chance of pain response for each 1 Gy/min increase in median dose rate (β, 0.26 [95% CI, −1.09 to 1.60]; *P* = .71). However, this relationship was not statistically significant. Heterogeneity measures (Tau^2^ = 0.04, I^2^ = 61.3%) and the test for heterogeneity (χ2 [Q] = 5.32, *P* = .07) continued to show moderate between-study heterogeneity.

#### Facial numbness

Facial numbness was assessed in 3 studies,^19^^,^^21^^,^^22^ which found nonsignificant relationships between high- and low-dose-rate groups. First, Arai et al^19^ observed a 14.5% incidence of new or worsening facial sensory loss at a dose rate ≤ 2.05 Gy/min, and 19.3% at a dose rate > 2.05 Gy/min (*P* = .407). Similarly, another study found a 16.9% incidence of new or worsening paresthesia or dysesthesia at a dose rate ≤ 2.05 Gy/min, and 14.5% with a dose rate > 2.05 Gy/min (*P* = .669).^19^ Second, Patel et al^22^ found a 16.1% incidence of facial numbness at a dose rate ≤ 2.05 Gy/min and 10% at a dose rate > 2.05 Gy/min (*P* = .34).^22^ Lastly, Barzaghi et al^21^ demonstrated a nonsignificant difference between high-dose-rate (>2.5 Gy/min) and hypoesthesia onset (*P* = .543).

### BED

The impact of BED on pain relief and sensory dysfunction was reported in 2019 patients in 4 studies.^23^, ^24^, ^25^, ^26^ The median BED_2.47_ was 2240 Gy (range, 1968-2613).^23^, ^24^, ^25^, ^26^

#### Pain relief

Among patients with a distal target location, Warnick et al^24^ demonstrated that a higher BED (BED_2.47_ ≥ 2100 Gy) is predictive of pain relief at 30 days (hazard ratio [HR], 1.46 [95% CI, 1.05-2.03]; *P* = .03) and at 1 year (HR, 1.20 [95% CI, 1.00-1.44]; *P* = .049). Similarly, Yang et al^23^ demonstrated that a higher BED is predictive of pain relief based on improvement in the Penn Facial Pain Scale-Revised across pain intensity items (*P* = .049), general activities of daily living interference items (*P* = .003), facial activities of daily living items (*P* = .047), and across all items (*P* = .025).^23^

Tang et al^26^ demonstrated an association between a higher BED (BED_2.47_ ≥ 2600.50) at the trigeminal root division level and increased pain relief based on improvement using the Barrow Neurological Institute pain relief classification (odds ratio [OR], 2.43 [95% CI, 1.21-4.88]; *P* = .013). Other authors also demonstrated a predictive relationship between a higher BED (BED_2.47_ ≥ 2535.48 Gy) and lower pain recurrence (HR, 0.61 [95% CI, 0.40-0.94]; *P* = .025).^24^ However, Tuleasca et al^25^ investigated freedom from pain at 1- and 2-year posttreatment and demonstrated no significant difference in relation to BED_2.47_, ranging from 1550 Gy to 2600 Gy.

#### Facial numbness

Tuleasca et al^25^ observed a significant relationship between higher BED and increased incidence of hypoesthesia, with less than 5% incidence at a lower BED (BED_2.47_ ∼1800 Gy) compared with 42% at BED_2.47_ ∼2600 Gy.^25^ Similarly, Yang et al^23^ demonstrated an increased incidence of facial numbness at a BED_2.47_ of 2004 to 2104 Gy (83.9%) compared with a lower BED_2.47_ of 1703 to 2003 Gy (40%) (*P* = .01).

Tang et al^26^ found that if more than 15.74% of the trigeminal root section adjacent to the root entry zone is treated with a BED_2.47_ of 1000 Gy, patients are more likely to experience new facial numbness (HR, 5.05 [95% CI, 3.86-8.89]; *P* < .0001). Similarly, for distal target locations, Warnick et al^24^ demonstrated that BED_2.47_ was correlated with the incidence of sensory dysfunction. However, BED_2.47_ was not significantly associated with sensory dysfunction in multivariable analyses that accounted for target location (distal vs proximal targets).^24^

## Discussion

In this study, we performed a meta-analysis to investigate the impact of dose rate and BED on TN pain response in GKRS. We observed that higher GKRS dose rates may result in greater pain relief.

Our findings can be compared with those of a meta-regression conducted by Yang et al,^23^ who demonstrated that each decrease in dose rate by 1.5 Gy/min corresponded to a 31.8% reduction in improvement in overall pain severity. The patient population in Yang et al’s^23^ retrospective analysis was very similar to the other included studies, with a similar proportion of female patients (60%) and a median dose rate (2.13 vs 2.2 Gy/min). However, our median BED_2.47_ was 2240 Gy, considerably greater than the 1961 Gy reported by Yang et al.^23^

Our narrative synthesis of BED also demonstrated that an increase in BED may be correlated with improved pain relief.^23^^,^^24^^,^^26^ We hypothesize that higher dose rates better disrupt the trigeminal nerve’s ability to transmit pain signals with a more potent radiation dose, a higher BED, and a shorter duration of time.^9^^,^^23^^,^^27^ This is consistent with the estimated low α/β ratio (ie, 2.47) of neural tissue.^28^ Thus, in clinical practice, we can adjust the dose prescription to maintain BED, ultimately delaying the need for expensive source replacement.

Our narrative synthesis of 3 cohorts investigating complications demonstrated no difference in sensory dysfunction rates across dose rates.^19^^,^^21^^,^^22^ However, BED was found to be either trending toward or associated with increased sensory dysfunction.^23^, ^24^, ^25^, ^26^

Strengths of this systematic review include a comprehensive search of clinical trials, explicit eligibility criteria, screening of studies, collection of data in duplicate to increase reliability, prospective protocol registration, and use of the GRADE approach to evaluate the certainty of the evidence. To the best of our knowledge, this study is the first systematic review and meta-analysis synthesizing the impact of dose rate on TN outcomes. Further, our evaluation of BED, in addition to dose rate, allows for the evaluation of factors associated with BED, including dose rate.^29^

Some limitations merit mention. First, the assumptions used to calculate BED varied between studies, contributing to considerable heterogeneity in our analysis. Of the 5 studies that calculated and reported BED, 3 studies^23^, ^24^, ^25^, ^26^ used the model described by Hopewell et al,^8^ and the other 2 studies^20^^,^^23^ reported the models by Nilsson and Thames^12^ and Thames.^13^ Further, while Yang et al,^23^ Tuleasca et al,^25^ Warnick et al,^24^ and Tang et al^26^ used the same parameters to calculate BED (α/β=2.47) and biexponential half-repair times of 0.19 hours and 2.16 hours, Balamucki et al^21^ used α/β=1.5 and a single repair time of 6.5 hours. Thus, cross-cohort comparisons are difficult, and a narrative synthesis was conducted in lieu of meta-analysis. Because of the small number of studies meeting the inclusion criteria and the heterogeneity of outcomes, we were able to pool data only on the degree of pain relief in TN. This demonstrates a gap in the literature, because we were limited in our ability to synthesize clinical and radiological outcomes in relation to GKRS dose rate across all lesion types.

Second, the included studies are largely retrospective in design, subject to the risk of selection and recall biases, potentially impacting the validity of the findings.^30^ Further, while our funnel plot did not detect publication bias in this study, we are cognizant that our analyses may be susceptible, because articles with negative findings (ie, lack of a demonstrated impact of dose rate on the outcomes) may be less likely to be published.^31^

## Conclusions

Higher dose rates and BED in GKRS may be associated with better pain relief among patients with TN. Although nonsignificant correlations between dose rate and facial numbness were identified in the narrative synthesis, increased BED was observed to be associated with increased toxicity. Further investigation of dose rate and BED in relation to pain relief and facial numbness outcomes is warranted to optimize thresholds for clinical application.

## Appendix Supplementary materials

Supplementary-material Image, application 1

## Footnotes

Supplementary material associated with this article can be found in the online version at doi:10.1016/j.adro.2025.101932.
