# Supplementary material for: Biologically Effective Dose and Dose Rate in Gamma Knife Radiosurgery for Trigeminal Neuralgia: A Systematic Review and Meta-Analysis
Source: Adv Radiat Oncol. 2025 Oct 23;11(1):101932. doi: 10.1016/j.adro.2025.101932 (PMC12681548; doi:10.1016/j.adro.2025.101932)

**SUPPLEMENTARY MATERIAL**

**Supplement 1.** Search strategy

# Query

1 (gamma knife or radiosurgery or GKRS or cobalt 60).mp.

2 (dose rate or dose-rate or biological effective dose).mp.

3 1 and 2 0

(gamma knife or radiosurgery or GKRS or cobalt 60).mp.

(dose rate or dose-rate or biological effective dose).mp.

1 and 2

**Supplement 2.** Risk of bias assessment using the CLARITY tool for cohort studies


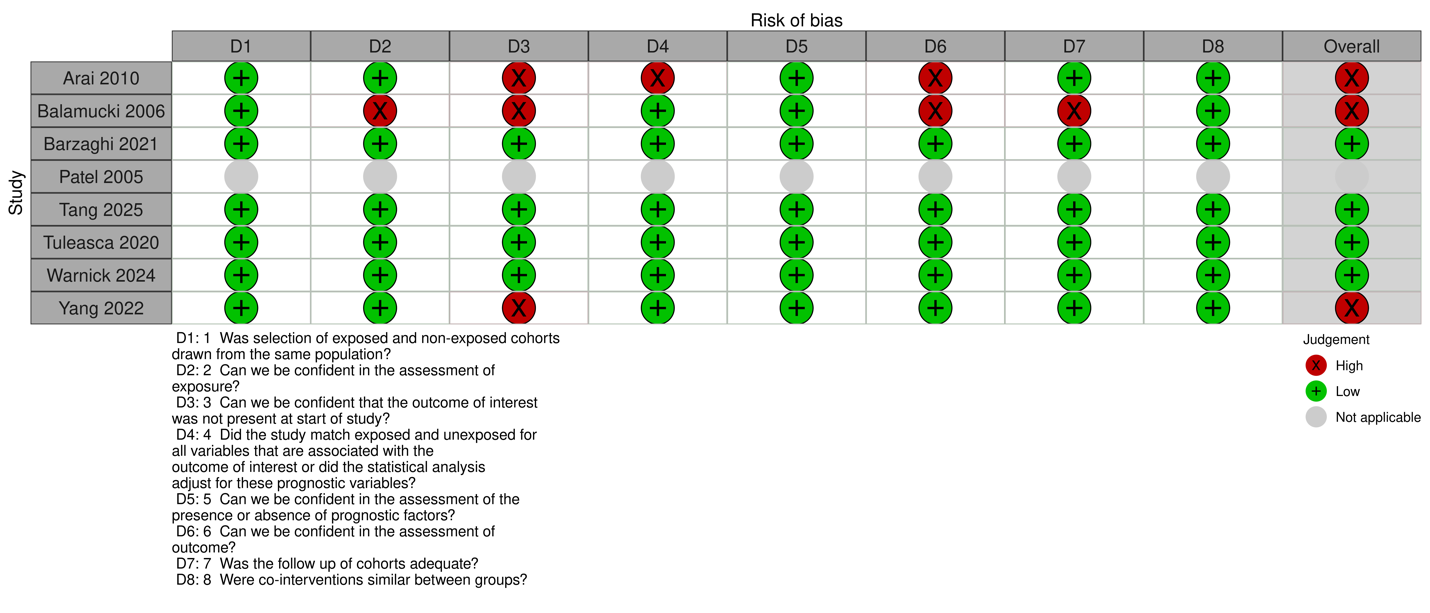


**Supplement 3.** GRADE evidence profile for degree of pain relief

| Outcome | No. of cohorts (no. of patients) | Risk of bias | Inconsistency | Indirectness | Imprecision | Publication bias | HR (95% CI) | Overall quality of evidence |
| --- | --- | --- | --- | --- | --- | --- | --- | --- |
| Trigeminal neuralgia pain relief | 4 (530 patients) | High^1^ | Low | Low | Low | Low | 1.36 (1.10-1.67) | Low |

^1^Risk of bias assessment using the CLARITY tool for cohort studies available in Supplement 2; Barzaghi 2021 (low risk), Yang 2022 (high risk), Patel 2005 (not a full-text article; cannot assess), Arai 2010 (low risk).

**Supplement 4.** Funnel plot to quantify publication bias


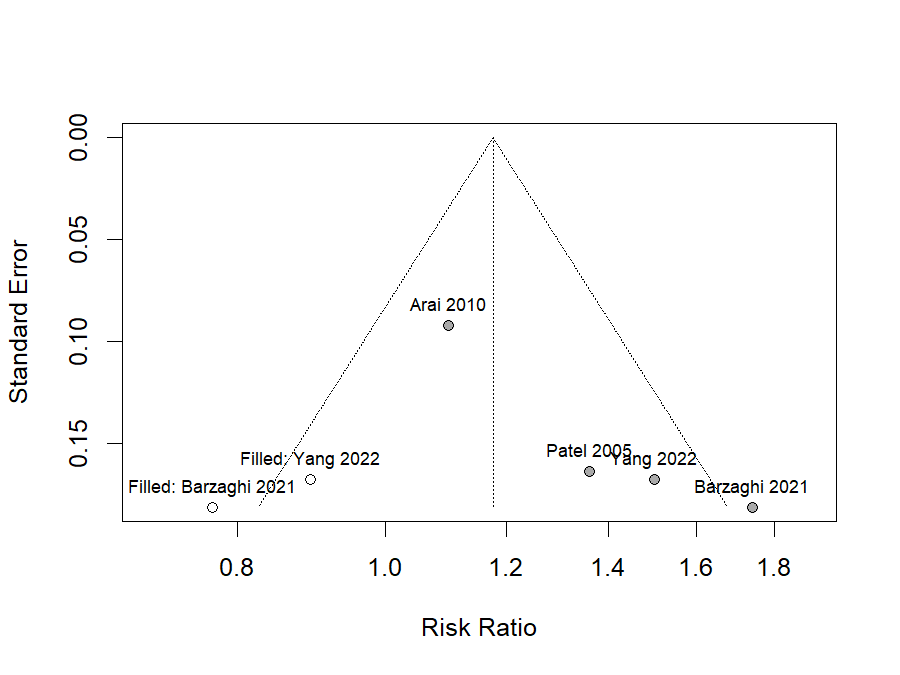

Supplement: Supplementary-material [file mmc1.docx]
